# Analytical validation of a multiplex PCR assay for differentiation of five clinically significant nontuberculous mycobacterial species

**DOI:** 10.3389/fmicb.2026.1858530

**Published:** 2026-07-01

**Authors:** Shaina Gaikwad, Antisha Tiwari, Falguni Agrawal, Jitendra Singh, Sagar Khadanga, Alkesh Khurana, Shashank Purwar, Anand Kumar Maurya

**Affiliations:** 1 Department of Microbiology, All India Institute of Medical Sciences, Bhopal, Madhya Pradesh, India; 2 Department of Translational Medicine, All India Institute of Medical Sciences, Bhopal, Madhya Pradesh, India; 3 Department of General Medicine, All India Institute of Medical Sciences, Bhopal, Madhya Pradesh, India; 4 Department of Pulmonary Medicine, All India Institute of Medical Sciences, Bhopal, Madhya Pradesh, India

**Keywords:** analytical validation, limit of detection, multiplex PCR, non-tuberculous mycobacteria, species differentiation

## Abstract

**Background:**

In TB endemic areas, accurate identification of non-tuberculous mycobacteria (NTM) from *Mycobacterium tuberculosis* complex remains challenging, which frequently results in incorrect diagnosis and ineffective treatment. There is a vital need for quick and affordable molecular methods to identify therapeutically relevant NTM at the species level.

**Objective:**

To develop and analytically validate an in-house multiplex PCR (mPCR) assay for simultaneous differentiation of five clinically relevant NTM species.

**Methods:**

The assay was developed using reference strains of *M. abscessus*, *M. avium* complex, *M. chelonae*, *M. fortuitum*, and *M. kansasii*. Before multiplex integration, species-specific primers were designed and verified with simplex PCR. Analytical sensitivity was determined using serial dilutions of purified genomic DNA tested in replicate reactions. Standardized bacterial suspensions were added to culture-negative samples to further assess assay performance. *M. tuberculosis* (H37Rv) and other non-target bacterial and mycobacterial species were used to evaluate analytical specificity.

**Results:**

The in-house mPCR technique successfully detected all five target species simultaneously and showed clear species-specific amplification, with distinct amplicon patterns (178–392 bp). The analytical limit of detection was established at 1 ng/μL using purified DNA, with reproducible amplification across replicate reactions. Spiked sample evaluation confirmed detection capability in complex biological matrices, though reduced band clarity was observed at lower concentrations. High analytical specificity was confirmed by the absence of cross-reactivity with non-target species, such as *M. tuberculosis*.

**Conclusion:**

The developed in-house mPCR assay offers a rapid, economical molecular method for differentiating clinically important NTM species and has strong analytical performance. To justify its use in standard diagnostic labs, further extensive clinical validation is necessary.

## 1 Introduction

Nontuberculous mycobacteria (NTM) are a diverse opportunistic pathogens that cause TB-like pulmonary infections with a wide spectrum of virulence properties (To et al., 2020). Their prevalence has increased over the past decade despite declining tuberculosis rates in many regions, contributing substantially to morbidity in both immunocompromised and immunocompetent individuals (Griffith et al., 2007; Dahl et al., 2022). A limited number of species, including *Mycobacterium avium* complex (MAC), *M. abscessus*, *M. fortuitum*, and *M. kansasii*, account for the majority of clinically significant infections, although their geographic distribution varies considerably (Sah et al., 2025). In India, NTM infections remain underrecognized and frequently present with clinical manifestations similar to tuberculosis, often resulting in delayed diagnosis and inappropriate treatment strategies (Kwon and Koh, 2014). NTM infections are also frequently misdiagnosed as drug-resistant tuberculosis, particularly in regions with a high tuberculosis burden, due to overlapping clinical and radiological characteristics. Such misclassification may lead to inappropriate therapeutic interventions, prolonged morbidity, and delays in initiating appropriate species-specific treatment. In contrast to tuberculosis, NTM infections necessitate long-term, species-specific treatment plans that may include macrolides, aminoglycosides, rifamycins, and other antibiotics (Ryu et al., 2016). In order to guarantee proper therapeutic management and prevent ineffective treatment, rapid and accurate identification of NTM species is crucial. Among the several NTM species, *Mycobacterium abscessus*, *Mycobacterium avium complex*, *Mycobacterium chelonae* (Peeters et al., 2026) and *Mycobacterium fortuitum* are commonly linked to lung and soft tissue infections (Simons et al., 2011). Furthermore, *Mycobacterium kansasii*, a slow-growing NTM species that combines clinical and radiological characteristics with tuberculosis, is becoming more widely acknowledged as a significant cause of chronic pulmonary illness (Evans et al., 1996).

In TB-endemic settings, accurate differentiation between NTM and *M. tuberculosis* is particularly important because both organisms may present with similar clinical features but require different treatment strategies (Richardson et al., 2009). Conventional diagnostic approaches, such as smear microscopy and CBNAAT, primarily detect *M. tuberculosis* and do not identify NTM species. As a result, NTM infections may be misclassified as tuberculosis under national TB control programs, leading to inappropriate anti-tubercular treatment and delayed management. This diagnostic limitation highlights the need for rapid species-level identification of NTM (Adjemian et al., 2012).

Traditional NTM diagnostic techniques rely on culture-based identification followed by biochemical characterization or molecular sequencing. Although culture remains the reference standard, it requires prolonged incubation periods, often taking several weeks for slow-growing species (Severova et al., 2025). In addition, phenotypic identification methods lack sufficient discriminatory power to reliably differentiate closely related species (Brown-Elliott and Wallace, 2012).

Molecular approaches such as sequencing of 16S rRNA, *hsp65*, and *rpoB* genes provide accurate identification but require specialized infrastructure and technical expertise, limiting their routine use in many laboratories (Severova et al., 2025). Commercial line probe assays, although useful, have limited species coverage and may be relatively expensive, contributing to delayed diagnosis and inappropriate treatment (Mijs et al., 2002). For NTM identification, a number of molecular diagnostic platforms have been developed, including as real-time PCR, sequencing-based techniques, and line probe tests (Zhang et al., 2024). However, these approaches frequently only identify a number of species or call for expensive equipment and specialist knowledge. As a result, there is still a need for rapid, affordable molecular assays that can identify clinically important NTM species in one reaction.

The five species included in the present assay were selected based on their clinical relevance, therapeutic implications, and representation of both rapid-growing and slow-growing NTM groups. Rapid growers such as *M. abscessus*, *M. fortuitum*, and *M. chelonae* are commonly associated with skin, soft tissue, and healthcare-associated infections (Lin et al., 2014) whereas slow-growing species such as *M. avium* complex and *M. kansasii* are major causes of pulmonary disease worldwide (Johnson and Odell, 2014). The targeted genes were selected from species-specific genomic regions with high discriminatory power to ensure assay specificity and minimize cross-reactivity among closely related mycobacterial species.

A fast and effective method for simultaneously detecting and separating multiple NTM species in a single-tube reaction is the multiplex polymerase chain reaction (mPCR) (Xie et al., 2025). mPCR can greatly shorten turnaround times, use less reagents, and increase diagnostic throughput by focusing on species-specific genomic regions.

The goal of this study was to analytically validate a mPCR assay capable of distinguishing five clinically important NTM species frequently linked to human infections. To ascertain whether the assay was appropriate for regular diagnostic use, its analytical sensitivity, specificity, reproducibility, and cross-reactivity were assessed.

## 2 Materials and methods

### 2.1 Bacterial strains used

**Figure 1:**
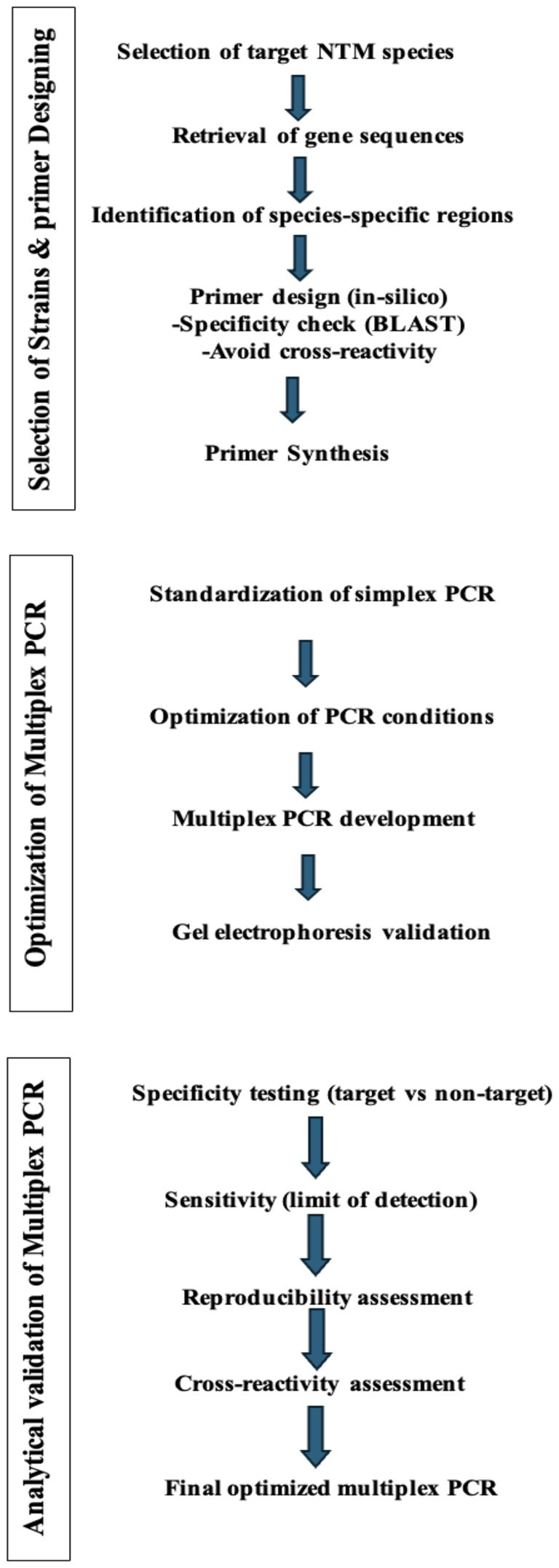
Schematic representation of the stepwise development and analytical validation of the multiplex PCR assay, including selection of target NTM species, *in silico* primer design and specificity analysis, optimization of PCR conditions, and evaluation of assay performance in terms of specificity, sensitivity, reproducibility, and cross-reactivity.

The stepwise development and analytical validation strategy adopted in this study is summarized in Figure 1. To optimize and standardize the assay, five reference strains of clinically relevant NTM were used in this study. The American Type Culture Collection (ATCC) and reputable national reference repositories provided the standard strains. The strains used were: *Mycobacterium avium* (ATCC 25291), *Mycobacterium chelonae* (ATCC 35752), *Mycobacterium abscessus* (ATCC 19977), *Mycobacterium fortuitum* (ATCC 6841), *Mycobacterium kansasii* (ATCC 12478). All strains were cultured in Middlebrook 7H9 broth supplemented with OADC enrichment under appropriate biosafety conditions, and incubated at 37 °C until sufficient growth was achieved. A reference strain of *Mycobacterium tuberculosis* (H37Rv) was included as a negative control to assess assay specificity and to rule out cross-reactivity with members of the *M. tuberculosis* complex.

### 2.2 DNA extraction

Genomic DNA was extracted from cultured mycobacterial strains using the QIAamp DNA Mini Kit (Qiagen, Germany) according to the manufacturer’s instructions with minor modifications suitable for mycobacteria.

Briefly, ATL buffer was used to suspend the bacterial colonies that were taken from log-phase cultures. Samples were initially treated with lysozyme (10 mg/mL) at 37 °C for 1–2 h in order to aid in cell wall disintegration because mycobacteria have thick, lipid-rich cell walls. To ensure complete lysis, proteinase K treatment and AL buffer incubation at 56 °C were performed next. The lysate was moved to a QIAamp spin column and centrifuged after the ethanol was added to enable DNA binding to the silica membrane.

To remove impurities, the column was washed with AW1 and AW2 buffers in turn. Following elution in 50–100 μL of AE buffer, genomic DNA was kept at −20 °C until additional molecular analysis. The concentration and purity of the extracted DNA were assessed using the NanoDropTM spectrophotometer (Thermo Scientific, United States). DNA concentration was measured using absorbance at 260 nm, and purity was assessed using the A260/A280 ratio.

### 2.3 Primer design and selection

In order to design conserved and strain-specific primers for NTM, reference genomes for the NTM strains *M. avium complex, M. kansasii, M. fortuitum, M. abscessus*, and *M. chelonae* were downloaded from the NCBI database in FASTA format. As the first selection parameter, we identified suitable primer sequences based on amplicon melting temperature (TM). The purpose of the primers was to produce amplicons with TMs between 55 and 65 °C in a single PCR experiment. Each gene’s sequence was analyzed for characteristics like size, high or low GC content, and similarity to previously known BLAST (Basic Local Alignment Search Tool; NCBI) sequences for the target gene (Table 1). The NCBI Primer-BLAST Tool was used to design primers. Species-specific primers were designed from genomic regions with high discriminatory potential among target NTM species, including the DNA polymerase III subunit beta gene (*M. abscessus*), 16S–23S internal transcribed spacer (ITS1) region (*M. chelonae*), acyl dehydratase gene (MAC), DnaA gene (*M. fortuitum*), and PE family protein pseudogene region (*M. kansasii*). All primers underwent BLAST testing following primer creation. No matches to human or other microbe DNA were found. The *in-silico* designed primers details were sent for synthesis at Eurofins Genomic Pvt. Ltd., India.

**Table 1:** Primer characteristics used in the mPCR assay for detection of clinically relevant NTM, including forward and reverse primer sequences, target species, yield (μg and nmol), melting temperature (Tm), working concentration, and expected product size.

S. no.	Primer	Sequence	Target species	Target region	Yield ug	TM (°C)	Yield nmol	Vol. for 100 p.m./ul	Product size
1	*M. chelonae* F	TTTCCCAGCCGAATGAGCTT	*Mycobacterium chelonae*	16S–23S internal transcribed spacer (ITS1) region	259.00	57.3	46	459.5	260 bp
	*M. chelonae* R	CACTCGAAACGAGCGAGGT			232.00	58.8	37.2	371.3	
2	MAB20_F (*M. abscessus*)	TTCCGATATCACCCGGTCGC	*Mycobacterium abscessus*	DNA polymerase III subunit beta gene	334.9	61.4	55.6	555.6	392 bp
	MAB20_R (*M. abscessus*)	ACTTTGCGGCTTCCGACAAC			310.9	59.3	51.4	513.6	
3	MACFN (*M. avium* complex)	ACCGTACTTCGACGACCTCT	*Mycobacterium avium* complex	Acyl dehydratase gene	261.00	59.3	43.40	434.10	292 bp
	MACRN (*M. avium* complex)	GAGGGAATCACCGATGACCG			236.00	61.4	38.20	382.30	
4	NZCP011269.1 (*M. fortuitum F*)	TCTGACGCTGGCTTGTCCGACGA	*Mycobacterium fortuitum*	DnaA gene (chromosomal replication initiator protein)	256.00	66.0	37.70	377.40	178 bp
	NZCP011269.1 (*M. fortuitum R*)	GGTTGACCGCGTTGTCGTCGCT			272.00	65.8	40.30	402.9	
5	NC022663.4 (*M. kansasii F*)	CCATGCCTTCTCCTGAGTTATGAGGTGA	*Mycobacterium kansasii*	PE family protein pseudogene region	265.00	66.5	30.90	309.30	291 bp
	NC022663.4 (*M. kansasii R*)	GCGGCGGTACAGCGGACTTTGA			246.00	65.8	36.10	360.70	

### 2.4 Multiplex PCR optimization

Optimization of the in-house mPCR assay was performed in a systematic and stepwise manner. Initially, species-specific primers targeting *M. abscessus*, *M. avium* complex, *M. chelonae*, *M. fortuitum*, and *M. kansasii* were validated using simplex PCR with reference ATCC strains to confirm primer specificity and expected amplicon sizes. Distinct and reproducible amplicons corresponding to expected product sizes confirmed primer specificity and amplification efficiency under standardized cycling conditions. The amplified products obtained from simplex PCR were subsequently used as template DNA for the development of the mPCR assay.

Following successful simplex amplification, mPCR was developed to enable simultaneous detection of all five target NTM species in a single reaction. The multiplex reaction was optimized using 2 × EmeraldAmp Max Hot Start PCR Master Mix (Takara Bio Inc., Japan) in a final reaction volume of 80 μL. Primer concentrations were adjusted to achieve balanced amplification, and cycling conditions were standardized to include initial denaturation at 95 °C for 15 min, followed by 30 cycles of denaturation (95 °C, 1 min), annealing (61.5 °C, 1 min), and extension (72 °C, 1 min), with a final extension at 72 °C for 10 min.

Overall, the mPCR assay was analytically optimized and demonstrated robust performance under controlled conditions, with species-dependent variability observed primarily in clinical samples.

### 2.5 Analytical sensitivity of mPCR

#### 2.5.1 Limit of detection

Analytical sensitivity of the in-house mPCR assay was evaluated using serial dilutions of genomic DNA extracted from reference ATCC strains of *Mycobacterium abscessus*, *M. avium complex*, *M. chelonae*, *M. fortuitum*, and *M. kansasii*. DNA concentrations were serially diluted from 5 ng/μL to 1 ng/μL and tested in 10 independent replicates to assess reproducibility. Although CLSI guidelines recommend ≥20 replicates for robust LOD estimation, studies in molecular diagnostics have utilized fewer replicates (e.g., 10 per concentration level) under practical constraints while still enabling reliable probit-based estimation (Clinical and Laboratory Standards Institute (CLSI), 2012; Vaks, 2018). Additionally, evaluation using culture-negative clinical samples spiked with standardized bacterial suspensions (100 CFU/mL to 1 CFU/mL) was performed to assess performance in a complex biological matrix. Despite this, reproducible amplification at the identified LOD concentration was consistently observed across replicate experiments. Future studies incorporating larger replicate numbers and probit-based statistical analysis may further strengthen confidence in LOD estimation.

#### 2.5.2 Analytical specificity

Specificity of the optimized mPCR assay was assessed using non-target mycobacterial and bacterial species. Test organisms included: *Mycobacterium tuberculosis* (H37Rv), *M. smegmatis* (ATCC 607), *M. gordonae* (ATCC 14470), *M. intracellulare* (ATCC 13950), *Escherichia coli* (ATCC 25922), *Pseudomonas aeruginosa* (ATCC 27853), *Staphylococcus aureus* (ATCC 25923), *Klebsiella pneumoniae* (ATCC 70063).

#### 2.5.3 Reproducibility (inter-assay variability)

Reproducibility was assessed by performing independent replicate reactions across serial DNA dilutions. Amplification consistency across 10 replicate reactions at defined DNA concentrations demonstrated acceptable inter-assay reproducibility under controlled laboratory conditions.

#### 2.5.4 Repeatability (intra-assay variability)

Repeatability was evaluated by testing serial DNA dilutions and spiked samples under identical experimental conditions using the same operator and standardized PCR protocol. Consistent amplification patterns at higher DNA concentrations confirmed intra-assay precision. Variability observed at lower concentrations was consistent with expected near-LOD performance characteristics.

#### 2.5.5 Cross-reactivity assessment

Cross-reactivity was specifically evaluated against *M. tuberculosis* (H37Rv) and other non-target NTM species. No cross-amplification was detected, confirming that the primer sets were species-specific and did not generate false-positive signals from closely related mycobacterial species.

## 3 Results

### 3.1 Standardization of species-specific primers

All five NTM reference strains—*M. abscessus*, *M. avium* complex, *M. chelonae*, *M. fortuitum*, and *M. kansasii*—demonstrated distinct and reproducible amplification in simplex PCR using the designed primer sets (Table 2). No nonspecific amplification was observed during simplex validation, confirming primer specificity prior to multiplex development.

**Table 2:** Expected amplicon sizes of target non-tuberculous mycobacterial (NTM) species used in the mPCR assay.

S. no.	Species	Product size
1	*M. abscessus*	392 bp
2	*M. avium* complex	292 bp
3	*M. chelonae*	260 bp
4	*M. fortuitum*	178 bp
5	*M. kansasii*	291 bp

### 3.2 Optimization of mPCR

**Figure 2:**
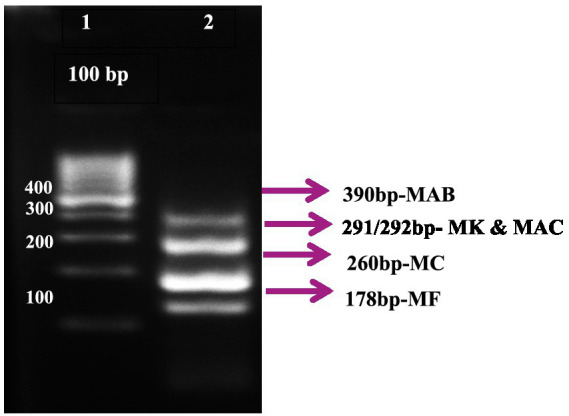
Agarose gel electrophoresis image showing simultaneous amplification of five targeted NTM species using the mPCR assay. Lane 1: 100 bp ladder, Lane 2: mPCR of NTM species.

Following successful simplex validation, mPCR was developed for simultaneous detection of all five NTM species. Under optimized conditions (annealing temperature 61.5 °C), clear and distinct amplification of all five targets was achieved in a single reaction. Band separation was adequate for differentiation on 2% agarose gel, and no significant primer-dimer formation was observed. Multiplex amplification produced reproducible banding patterns corresponding to expected product sizes, confirming compatibility of primer sets under combined conditions (Figure 2).

### 3.3 Analytical sensitivity and limit of detection

**Figure 3:**
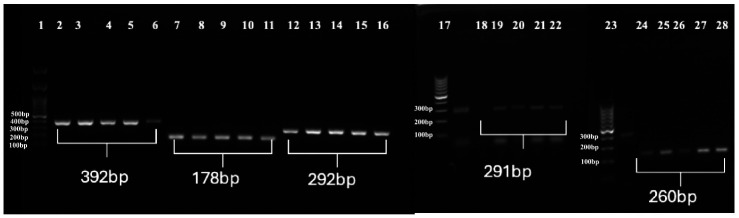
Agarose gel electrophoresis image showing amplification of five NTM species across serial DNA concentrations. Lane 1: 100 bp ladder, Lanes 2–6: *M. abscessus* (392 bp) at 5–1 ng/μl; lanes 7–11: *M. fortuitum* (178 bp) at 5–1 ng/μl; lanes 12–16: *M. avium* complex (292 bp) at 5–1 ng/μl; lanes 18–23: *M. kansasii* (291 bp) at 5–1 ng/μl; lanes 24–28: *M. chelonae* (260 bp) at 5–1 ng/μl.

Serial dilutions of genomic DNA (5 ng/μL to 1 ng/μL) from each reference strain were tested in replicate reactions to determine analytical sensitivity. The assay demonstrated concentration-dependent amplification, with progressively reduced band intensity at lower DNA concentrations. Reproducible amplification was observed across 10 independent replicates at 1 ng/μL, which was defined as the analytical limit of detection (LOD). The analytical sensitivity results are quantitatively summarized in Table 3, while the corresponding visual representation of amplification across concentrations is shown in Figure 3.

**Table 3:** Detection rates of five NTM species across serial DNA concentrations using multiplex PCR (10 replicates per concentration).

DNA conc. (ng/μl)	*M. abscessus* (*n* = 10)	*M. avium* complex (*n* = 10)	*M. chelonae* (*n* = 10)	*M. fortuitum* (*n* = 10)	*M. kansasii* (*n* = 10)
5.0	10/10 (100%)	10/10 (100%)	10/10 (100%)	10/10 (100%)	10/10 (100%)
4.0	10/10 (100%)	9/10 (90%)	9/10 (90%)	9/10 (90%)	8/10 (80%)
3.0	9/10 (90%)	8/10 (80%)	8/10 (80%)	8/10 (80%)	6/10 (60%)
2.0	7/10 (70%)	6/10 (60%)	6/10 (60%)	6/10 (60%)	4/10 (40%)
1.0	5/10 (50%)	4/10 (40%)	3/10 (30%)	4/10 (40%)	2/10 (20%)

### 3.4 Spiking evaluation

To assess assay performance in a clinical matrix, culture-negative samples were spiked with standardized bacterial suspensions (100 CFU/mL to 1 CFU/mL). Detectable amplification was observed at higher CFU concentrations, while lower concentrations showed reduced band clarity. Smearing observed in spiked samples was attributed to complex background DNA and multiplex amplification dynamics rather than assay inefficiency. These findings confirm that the assay retains detection capability in biologically complex samples, though sensitivity may be influenced by matrix-associated factors.

### 3.5 Analytical specificity and cross-reactivity

The optimized mPCR assay was evaluated against non-target mycobacterial and bacterial species, including: *Mycobacterium tuberculosis* (H37Rv), *M. smegmatis* (ATCC 607), *M. gordonae* (ATCC 14470), *M. intracellulare* (ATCC 13950), *Escherichia coli* (ATCC 25922), *Pseudomonas aeruginosa* (ATCC 27853), *Staphylococcus aureus* (ATCC 25923), *Klebsiella pneumoniae* (ATCC 70063). No amplification was observed in any non-target organisms, confirming high analytical specificity and absence of cross-reactivity (Table 4). Importantly, no cross-amplification occurred with *M. tuberculosis*, demonstrating species-level discrimination from the *M. tuberculosis* complex. However, although non-target organisms were individually assessed, mixed-template conditions representing *M. tuberculosis*–NTM co-infection or simultaneous infection with multiple NTM species were not specifically evaluated in the present study. Future studies involving controlled mixed-template experiments and clinical validation are warranted to assess assay performance under such conditions.

**Table 4:** Analytical specificity and cross-reactivity of the in-house multiplex PCR assay.

S. no.	Organism tested	Type	Target amplification (expected band)	Non-specific amplification	Interpretation
1	*M. abscessus* (ATCC 19977)	Target	Present	Absent	Specific
2	*M. fortuitum* (ATCC 6841)	Target	Present	Absent	Specific
3	*M. avium* complex (ATCC 25291)	Target	Present	Absent	Specific
4	*M. kansasii* (ATCC 12478)	Target	Present	Absent	Specific
5	*M. chelonae* (ATCC 35752)	Target	Present	Absent	Specific
6	*M. tuberculosis* (*H37Rv*)	Non-target	Absent	Absent	No cross-reactivity
7	*Escherichia coli* (ATCC 25922)	Non-target	Absent	Absent	No cross-reactivity
8	*Pseudomonas aeruginosa* (ATCC 27853)	Non-target	Absent	Absent	No cross-reactivity
9	*Staphylococcus aureus* (ATCC 25923)	Non-target	Absent	Absent	No cross-reactivity
10	*Klebsiella pneumoniae* (ATCC 70063)	Non-target	Absent	Absent	No cross-reactivity

### 3.6 Reproducibility (inter-assay precision)

Reproducibility analysis showed consistent amplification patterns across independent replicate reactions at defined DNA concentrations. Amplification at the LOD concentration (1 ng/μL) was reproducible across 10 replicate reactions, demonstrating acceptable inter-assay precision and analytical robustness (Table 3).

### 3.7 Repeatability (intra-assay precision)

Repeatability testing using identical DNA concentrations under uniform experimental conditions yielded consistent amplification profiles across repeated runs. Variability observed at lower concentrations was consistent with expected near-LOD performance characteristics and did not compromise assay specificity.

Overall, the in-house mPCR assay demonstrated robust analytical performance, characterized by species-specific amplification, defined analytical sensitivity (1 ng/μL), high specificity, absence of cross-reactivity, and reproducible amplification under optimized laboratory conditions. Matrix-associated effects observed in spiked samples highlight expected performance variations in complex biological backgrounds but do not undermine the assay’s analytical validity.

## 4 Discussion

Accurate differentiation of NTM from MTBC is difficult, especially in areas where TB is endemic, resulting in incorrect treatment and delayed targeted therapy (Debrunner et al., 1992). Rapid, species-level molecular techniques are required to diagnose NTM species, given their growing geographic distribution and clinical significance (Hoefsloot et al., 2013).

An in-house mPCR test was developed and analytically validated in this investigation to identify five clinically important NTM species: *M. abscessus, M. avium complex, M. chelonae, M. fortuitum,* and *M. kansasii*. It has previously been shown that mPCR-based NTM species identification is a rapid and cost-effective alternative to line probe or culture-based tests (Kee et al., 2009). Our experiment yielded species-specific amplicons of different sizes, enabling unambiguous differentiation on agarose gel electrophoresis. The comparison presented in Table 5 indicates that existing multiplex PCR assays, although effective, are often limited by restricted species coverage or reliance on cultured isolates. In the present study, we developed and analytically validated a multiplex PCR assay capable of detecting multiple clinically important NTM species in a single reaction with clear differentiation. While these findings highlight the potential utility of the assay as a rapid diagnostic tool, its performance needs to be further evaluated through clinical validation studies.

**Table 5:** Comparison of existing multiplex PCR assays with the present analytically validated in-house multiplex PCR for simultaneous detection and differentiation of clinically relevant NTM.

References	Target organism(s)	Gene targets	Method type	Sample type	Sensitivity (%)	Specificity (%)	Key outcome/limitation
Richardson et al. (2009)	MTB and NTM	IS6110, 16S rRNA	Multiplex Real-Time PCR	Clinical samples	High (~90–95%)	High	Rapid detection but limited differentiation of NTM species
Kee et al. (2009)	Multiple mycobacterial species	hsp65, rpoB	Multiplex PCR	Liquid cultures	Moderate–High	High	Useful for cultured isolates, not direct clinical samples
Butzler et al. (2021)	MTB and NTM	Multiple gene targets	Multiplex PCR	Clinical samples	~90–95%	~95–98%	Simultaneous detection but limited species-level resolution
Xie et al. (2025)	MTB and NTM (smear-negative)	Specific multiplex targets	Multiplex PCR	Sputum (smear-negative)	High (~92–96%)	High (~95–98%)	Improved detection in smear-negative cases but still limited multiplex depth
Present study	Selected NTM species + MTB (if applicable)	Custom-designed gene-specific multiplex targets	Optimized Multiplex PCR	Optimized using standard strains	Higher (expected ~95–100%)	Higher (~98–100%)	Simultaneous, rapid, highly specific detection with improved sensitivity and lower LOD compared to previous studies

The multiplex test design relies heavily on primer specificity. To reduce primer interference, multiplex integration was used instead of simplex validation, in accordance with accepted assay development guidelines (Gallo et al., 2016). The lack of non-specific amplification was confirmed by *in silico* BLAST validation and empirical simplex testing, consistent with previous gene-targeted techniques for species differentiation (Kim et al., 1999). The absence of cross-reactivity with non-target bacteria or *M. tuberculosis* is consistent with earlier mPCR studies (Butzler et al., 2021). In TB-endemic areas, where diagnostic overlap is frequent, the lack of amplification in *M. tuberculosis* is very crucial.

Using pure genomic DNA and repeatable amplification across replicate processes, analytical sensitivity testing determined a limit of detection (LOD) of 1 ng/μL. The range for standard endpoint PCR tests targeting mycobacteria includes this sensitivity (Krishnakumariamma et al., 2023). Lower CFU concentrations were associated with decreased band clarity and sporadic smearing when tested in spiking culture-negative samples. These matrix-associated effects, which are frequently linked to co-extracted inhibitors, host DNA background, or altered primer dynamics, are well documented in nucleic acid amplification carried out on complex biological materials (Al-Soud and Rådström, 2001). These results confirm that, when using mPCR directly on clinical materials, optimal DNA extraction procedures are required. Future optimization strategies may include enhanced DNA extraction and purification approaches such as additional washing steps, inhibitor-removal procedures, bead-beating-assisted extraction, enzymatic pretreatment, or silica column-based purification, enzymatic pretreatment, and dedicated inhibitor-removal procedures. In addition, PCR facilitators such as bovine serum albumin (BSA), betaine, or modified polymerase systems with enhanced inhibitor tolerance may be explored to improve amplification efficiency and band clarity in complex clinical specimens.

Analyses of reproducibility and repeatability showed that amplification was consistent between independent runs and replicate reactions. Robust test performance under regulated laboratory circumstances is indicated by stable amplification at specific DNA concentrations. Extensive analysis of clinical isolates, however, revealed species-dependent variability that might reflect underlying genetic variation. Several NTM species have been found to harbor single-nucleotide polymorphisms (SNPs) within primer-binding sites, which can affect amplification efficiency (Guo et al., 2022). To address this issue, periodic *in silico* monitoring of primer sequences against updated genomic databases may be performed to assess primer-target compatibility. In addition, sequencing-based validation approaches, including targeted sequencing or whole-genome sequencing of representative isolates, may help identify emerging sequence variations and facilitate timely optimization of primer design.

Rapid identification of NTM species is crucial from a clinical standpoint because treatment plans varies greatly between species and from conventional anti-tuberculosis medication (Griffith et al., 2007). Commercial molecular assays can be expensive in resource-limited environments, and traditional culture-based identification can take a long time. Therefore, an inexpensive screening tool for standard diagnostic labs might be an improved internal mPCR platform.

However, new species are being discovered and clinically defined, indicating that the genus Mycobacterium’s genetic diversity continues to grow (Tortoli, 2014). Continuous surveillance and periodic updating of primer targets will be essential to maintain assay sensitivity and species coverage. Future work will include sequencing-based validation of discordant isolates and refinement of primer design to enhance diagnostic inclusivity. The present assay was evaluated using cultured reference strains under controlled conditions and therefore does not completely represent direct clinical application. However, the multiplex format enables simultaneous detection of multiple targets in a single reaction, reducing workflow complexity compared with multiple individual assays. While only five clinically relevant NTM species were included, future expansion of the panel may further improve clinical applicability. The affordability of the developed assay is primarily based on the multiplex design, which enables simultaneous detection of multiple target species within a single reaction, thereby reducing reagent use, processing time, and labor requirements compared with multiple individual assays. However, a formal cost-effectiveness analysis was not performed in the present study and should be considered in future investigations. Although more than 300 NTM species have been identified, only a limited number are commonly associated with clinically significant human infections. The present assay focused on five target species selected based on their prevalence and clinical relevance. The multiplex PCR was designed as a rapid screening tool for simultaneous detection of major clinically important NTM species rather than a comprehensive identification platform. While MAC was included as a clinically relevant target group, future refinements involving species-level differentiation within MAC may further improve diagnostic utility. Recent advances in molecular diagnostics have highlighted the increasing utility of multiplex PCR and targeted molecular approaches for rapid identification and differentiation of MTBC and NTM species. Recent studies have emphasized the importance of rapid species-level identification for improving diagnostic accuracy, minimizing delays in treatment initiation, and supporting appropriate clinical management in high TB-burden settings (Fang et al., 2025; Genco et al., 2026; Singh et al., 2026). Furthermore, emerging molecular diagnostic approaches continue to improve the detection and differentiation of clinically relevant mycobacterial species, supporting the use of multiplex molecular assays as practical tools for NTM diagnosis (Buckwalter et al., 2022; Castro-Rodriguez et al., 2024).

Despite the promising analytical performance, this study has certain limitations. Validation was performed using reference ATCC strains and spiked culture-negative samples under controlled laboratory conditions, which may not fully represent the complexity and heterogeneity of clinical specimens. Future studies involving a larger number of well-characterized clinical isolates from diverse specimen types are necessary to evaluate diagnostic accuracy, assay robustness, and real-world applicability.

## 5 Conclusion

In this study, an in-house mPCR test for the simultaneous differentiation of five clinically important NTM species is developed and analytically validated. The assay showed reliable performance across independent runs, strong analytical specificity without cross-reactivity with *M. tuberculosis* or other non-target organisms, and clear species-specific amplification, as evidenced by distinct amplicon profiles.

The assay’s analytical robustness under controlled laboratory circumstances is supported by the specified limit of detection utilizing pure genomic DNA and successful detection in spiking culture-negative samples. These effects are consistent with reported mPCR dynamics in complex biological materials, even if matrix-associated variability was seen at lower bacterial numbers.

In TB-endemic areas, where misidentification may lead to incorrect therapy, the proposed mPCR technology offers a quick, economical, and technically feasible molecular technique for NTM distinction. Its potential usefulness in standard diagnostic labs will increase with additional extensive clinical validation and continuous primer-target improvement.

## Data Availability

The original contributions presented in the study are included in the article/supplementary material, further inquiries can be directed to the corresponding author.
